# Editorial: High altitude medicinal plants and their bioactive compounds for the prevention of oxidative stress-induced diseases and disorders

**DOI:** 10.3389/fphar.2023.1257018

**Published:** 2023-08-11

**Authors:** Prabhakar Semwal, Abdur Rauf, Jesus Simal-Gandara

**Affiliations:** ^1^ Department of Biotechnology, Graphic Era (Deemed to be University), Dehradun, India; ^2^ Research and Development Cell, Graphic Era Hill University, Dehradun, India; ^3^ Department of Chemistry, University of Swabi, Khyber Pakhtunkhwa, Pakistan; ^4^ Analytical Chemistry and Food Science Department, Faculty of Science, Universidade de Vigo, Nutrition and Bromatology Group, Vigo, Spain

**Keywords:** high altitude, Himalaya, bioactive compounds, health promoting effects, oxidative stress, biological applications

Oxidative stress is a biochemical process occurring in cells and tissues due to the abnormal accumulation and production of reactive oxygen species (ROS)/free radicals, in which the endogenous antioxidants are unable to neutralize these free radicals and cause oxidative damage to proteins, lipids, DNA, etc. of the cells (Pizzino et al., 2017; Sharifi-Rad et al., 2020). If not managed properly it can accelerate the aging process and can be responsible for causing acute pathologies like trauma, stroke, etc., as well as many chronic and degenerative diseases and disorders (Vona et al., 2021).

Bioactive compounds are extra-nutritional constituents, generally found in different types of foods, fruits, vegetables, and grains, which provide health benefits beyond their basic and traditional nutritional value (Zhao et al., 2015). A large number of bioactive constituents have been isolated from botanicals, and these botanicals have been utilized from antiquity for their therapeutic abilities.

High-altitude medicinal plants are known for their ethnobotanical importance and have been used by various communities around the world for the treatment of various diseases and disorders due to their healing properties. The diverse geographical and climatic condition including biotic and abiotic factors leads to the production of novel bioactive metabolites in these plants (Heinrich et al., 2021; Semwal et al., 2022). In the past few years, many potential metabolites such as phenolic acids, alkaloids, flavonoids, volatile oils, glycosides, etc. have been discovered from natural sources for their antimicrobial, cardioprotective, anti-inflammatory, anticancer and chemoprotective activities. There is considerable evidence to suggest that these phytochemicals are beneficial to health on an epidemiological and clinical level (Chan et al., 2023; Du et al., 2023; Rahaman et al., 2023; Xu et al., 2023; Zhou et al., 2023; Zhu et al., 2023).

The present Research Topic aims to provide a platform for current research and evidences available about the role of High-altitude medicinal plants, their chemical components, and its role in reducing the risk of diseases and disorders related to oxidative stress. Within this dedicated edition, the guest editorial board was overwhelmed to receive a remarkable series of scientific reports from numerous regions around the world. In all 16 original research and review articles were submitted and after the critical evaluation and peer review process total 6 articles (02 Original research article, 01 Systematic review and 03 Review articles) have been approved for the publication in this special edition.

Limonin, a triterpenoid molecule that is typically present in citrus fruits, exhibits a diverse range of pharmacological properties, including antioxidant, antiviral, anti-inflammatory, anti-cancer, and liver protecting qualities. The first research article by Li et al. describes the protective effects of limonin compound and their molecular mechanism against non-alcoholic fatty liver disease by means of *in-vitro* and *in-vivo* models. In this investigation, zebrafish larvae were treated with thioacetamide to create a model of NAFLD, and the larvae received limonin treatment parallelly for 72 h. According to the findings, limonin dramatically decreased the formation of lipid bodies in liver and downregulated the levels of sterol regulatory element binding protein 1 (SREBP-1), two lipogenic transcription factors associated with NAFLD and fatty acid synthase. The study also showed that limonin inhibited the invasion of macrophages and reduced the expression levels of the pro-inflammatory mediators including IL-1beta, IL-6 and TNF-alpha, which are released by macrophages. Furthermore, limonene can reverse glutathione depletion and reactive oxygen species accumulation by regulating the NRF2/HO-1 signalling pathway in the liver. In conclusion, this study revealed that limonin has pharmacological effects in the treatment of non-alcoholic fatty liver disease by reducing oxidative stress, lipid accumulation, and inflammation induced by pro-inflammatory chemokines and macrophage infiltration Li et al.

Paeonol, a phenolic compound present in various plant species, is known to have several pharmacological activities, including antitumor, anti-inflammatory, neuroprotective, cardioprotective, nephroprotective activities. The second research article demonstrated the *in-vivo* anti-diabetic activity of paeonol against diabetic retinopathy. In this study, diabetes was induced using streptozotocin (55 mg/kg, i.p.) in male Sprague Dawley rats, and diabetic rats were administered with paeonol at doses between 50 and 200 mg/kg per day for the next 4 weeks after the initial 4-week treatment period. Different parameters such as retinal physiology, histopathology, biochemical and oxidative stress were recorded. The electroretinogram recording of paeonol-treated rats exhibited a substantial improvement in the a-wave amplitude, b-wave amplitude, a-wave latency, and b-wave latency (p˂0.001) at 15 cd s/m^2^ when compared with control (diabetic animals). Additionally, paeonol treated animals exhibited a substantial drop in the plasma glucose level, and aldose reductase, sorbitol dehydrogenase and lactate dehydrogenase level compared to diabetic control animals. According to the study’s findings, paeonol can be used as a management strategy for diabetic retinopathy (Adki and Kulkarni).

Worldwide, cardiovascular diseases (CVD) are among the leading causes of death. In a systematic review, Zeng et al. reported the cardioprotective effects of curcumin on myocardial ischemia/reperfusion injury using preclinical meta-analysis in animal studies. This meta-analysis comprised 37 studies with a total of 771 animals, with technique quality values ranging from 4 to 7. The study suggests that curcumin treatment significantly reduced the extent of myocardial infarction, and also decreased the serum inflammatory cytokines and the myocardial apoptotic index Zeng et al.

The first review report on protective effects of salidroside’s against different ischemia-related diseases with its possible molecular mechanism has been reported by Han et al. Salidroside, one of the main active components of *Rhodiola* species has been suggested to treat ischemia and ischemic damage by increasing the rate of cell survival and angiogenesis and reducing oxidative stress and inflammation. This article describes recent advances and research progress on salidroside for the treatment of ischemic diseases, including ischemic heart disease, ischemic acute kidney injury, cerebral ischemia, liver ischemia, etc., Han et al.

Gynaecological malignancies, such as ovarian, cervical, and endometrial cancers, have had a significant negative impact on women’s health due to concealed illnesses, incorrect diagnosis, and high recurrence rates. Li et al. have investigated the molecular mechanisms of the beneficial effects of natural polysaccharides in treating gynecological cancer. In this review article, the role of natural polysaccharides, preparation of new dosages, available scientific evidences have been included Li et al.

Fordjour et al. explore the therapeutic potential of *Cannabis sativa*. In this articles, scientific information has been collected on different aspects such as ecology, chemical compounds, ethnomedicinal applications, biological applications, industrial applications and toxicological studies.

Overall, taken together the data available in this special edition clearly indicated the significant pharmacological effects of naturally-occurring bioactive constituents in multiple affections, with promissory data being increasingly published. However, despite these advances, it is still necessary to clarify the effects of single and multiple doses of drugs, pharmacokinetics, efficacy, their toxicity, pharmacodynamics, and safety profiles, as well as their mechanism of action, for a clearer understanding of their therapeutic properties and to establish stronger evidence-based medicine.
